# Assessment of Hepatitis B Viral Infection as a Predictor of Hepatic Enzymes and Compounds Alteration among Antenatal Patients

**DOI:** 10.3390/medsci5040024

**Published:** 2017-10-18

**Authors:** Olatunji Ayodeji Abulude, Ismai’la Ahmed, Farouk Umar Sadisu

**Affiliations:** 1 Department of Biological Sciences, Faculty of Science, Nigeria Police Academy, Wudil, P.M.B. Kano 3474, Kano State, Nigeria; 2 Department of Microbiology, Kano University of Science and Technology, Wudil, P.M.B. Kano 3244, Kano State, Nigeria; ismahmed2k5@yahoo.com (I.A.); sadisufu69@gmail.com (F.U.S.)

**Keywords:** hepatitis B virus, prevalence, hepatic enzymes, liver function tests, predictor, antenatal patients

## Abstract

Worldwide, hepatitis B viral (HBV) infection continues to be a major public health issue. The study was aimed at assessing HBV infection as a predictor of hepatic enzymes and compounds alteration among antenatal patients in Kano State, Nigeria. Sera were screened for HBV markers using immunochromatograhy and ELISA. Serum levels of alkaline phosphatase (ALP), asphatate aminotransferase (AST), alanine aminotransferase (ALT), albumin and bilirubin were also determined. Out of the 160 patients, 6.9% and 31.3% tested positive for HBsAg and HBcAb, respectively. None tested positive for HBeAg. These markers also appeared in other combinations. Of the HBsAg seropositives, 72.7% showed abnormal levels of both AST and ALP, 36.7% showed abnormal levels of both total and direct bilirubin, 9.1% showed abnormal levels of albumin, and none showed abnormal levels of ALT. HBsAg seropositivity shows significant association with ALP elevation (*p* = 0.02).The study revealed that few subjects (1.3%) that tested positive for HBsAg and HBeAb with normal ALT were in the inactive carrier phase of chronic hepatitisand6.9% that were seronegatives for all HBV markers equally had altered hepatic enzymes. The presence of HBeAg in the serum during HBV infection seems to cause a marked elevation of ALT level, while the reverse happens if HBeAg is absent. HBV infection can alter levels of hepatic enzymes and compounds and thus serve as one of its predictors, however; pregnancy can also lead to some of these alterations, which makes it difficult to establish the origin of these alterations among antenatal patients.

## 1. Introduction

Hepatitis B virus (HBV) is the leading cause of hepatocellular carcinoma (HCC) and cirrhosis worldwide [1]. It is estimated that 44% of cirrhotic disease and 47% of HCC cases in sub-Sahara Africa are attributed to HBV infection [2]. Hepatic diseases account for 7.9% of medical admissions in Nigeria, with primary hepatic cancer and cirrhosis accounting for 44.3% and 20.4%, respectively. HBV is the second main cause of these conditions with the prevalence of 49.4% after alcohol consumption (52.1%) [3]. There is an increasing trend of HBV infection in Nigeria [4], so the risk of contracting HBV in Nigeria is substantial not only due to low vaccination rates but also because as many as 75% of the population will be exposed [1]. Acute hepatitis in pregnancy has been shown to cause jaundice and can also induce premature labor and prematurity with the associated complications. Likewise, women with chronic hepatitis or cirrhosis exhibit a higher risk of fetal loss during pregnancy. In developed countries, pregnant women are routinely screened for HBV infection. However, in developing countries, the case is different, as many women are not screened during pregnancy. The HBV status of the mother must be known before parturition in order to prevent HBV transmission from mother to child [5,6]. Different serological markers of diverse clinical and epidemiological importance are presented once infected with HBV. Depending on the stage and natural history of the disease, these serological markers can occur individually or in different combinations [7]. Biomarkers are equally useful in the evaluation and assessment of hepatic function and disease severity because HBV infection may alter the serum levels of certain hepatic enzymes and compounds such as alkaline phosphatase (ALP), asphatate aminotransferase (AST), alanine aminotransferase (ALT), bilirubin, and albumin [8]. The elevation of these enzymes and proteins above their upper reference limits are said to be abnormal except for serum albumin, which usually falls below its reference limit when it is abnormal. Reference ranges for the same enzymes and tests differ among laboratories and geographical locations [9]. As hepatic disease becomes severe, aminotransferases are usually elevated but may not correlate well with the disease. However, as the disease progresses, the serum level of albumin, bilirubin, and prothrombin time usually become altered, while reduction in platelet counts is usually an unreliable prognostic sign. Marked elevation in serum ALT with acute flare-up may be seen in patients with chronic hepatitis [10]. Normally during pregnancy, changes are often seen in hepatic biochemical profile. Usually, there is elevation in the level of serum ALP, and this elevation may be up to 2–4 times the normal baseline level. This is because the placenta produces additional ALP during pregnancy, while the serum albumin usually drops, and this is attributed to the total plasma volume. However, the serum levels of AST, ALT, and bilirubin usually remain normal, and any elevation seen should be investigated [11]. AST and ALT are often released into the bloodstream once there is hepatocellular damage, so ALT serum level elevation correlate more with hepatic injury. Sometimes the ratio of ALT to AST can also help define the patterns of a disease [12]. The initial non-specific testing of HBV infection is done by assessing liver function biochemically, while the specific diagnosis of HBV infection involves the evaluation of specific HBV serological markers, which include certain antigens and antibodies [13].

Generally, liver function tests are helpful to assess the severity and predict the outcome of certain liver diseases such as viral hepatitis. The aim of this study was to assess hepatitis B viral infection as a predictor of alteration in hepatic enzymes and compounds among the study group.

## 2. Materials and Methods

### 2.1. Study Area

This study was conducted in three secondary health care centers in Kano South Senatorial District, Kano State, Nigeria. They include General Hospitals in Sumaila, Wudil, and Gaya. These hospitals have antenatal clinics that provide antenatal care for pregnant women in these localities twice weekly on Mondays and Thursdays. These clinics have an average daily attendance of about 250 patients.

### 2.2. Study Design

The study was multicentered, hospital-based, and cross-sectional. 

### 2.3. Ethical Consideration

Ethical approval was obtained from the Ethics Committee, Operational Research Advisory Council of the Kano State Ministry of Health (No. MOH/Off/797/T.I./155). Informed consent of each participant was obtained prior to sample collection by the issuance of a consent form.

### 2.4. Inclusion and Exclusion Criteria

The inclusion criteria were all pregnant women attending the antenatal clinics of these hospitals that consented to taking part in the study. Those who did not register with the antenatal clinic of these hospitals and those who declined to take part in the research were excluded.

### 2.5. Sample and Data Collection

One hundred and sixty antenatal patients were sampled at the three study areas. Systemic random sampling was used by selecting every fifth woman on the waiting line of the antenatal clinics. In total, 160 blood samples were collected. Five milliliters (5 mL) of blood were collected aseptically from each patient into a plain bottle and transported to the laboratory immediately for analysis. Each sampling bottle was properly labeled for easy identification. Samples that were not analyzed immediately were refrigerated between 2 and 8 °C for 72 h after collection. Samples were stored at −20 °C for longer period [14,15,16]. Information regarding the vaccination status and alcohol consumption of the participants was also collected.

### 2.6. Processing of Blood Sample

About 1 mL of the whole blood was set aside for use immediately for rapid test, while the remaining 4 mL were allowed to stand at room temperature for 1 h to effect clotting and then centrifuged at 2500 rpm for 10 min in a vacutainer. The sera were then separated into a plain blood collection container and stored at −20 °C until needed [17].

### 2.7. Analysis of Blood Samples

#### 2.7.1. Serology Tests

Whole blood samples were subjected to rapid test using immunochromatographic assay for the qualitative detection of hepatitis B markers in serum. Micropoint kit (Micropoint Bioscience Ltd., Santa Clara, CA, USA) was used for the test. It is a combo kit for the identification of HBsAg, HBsAb, HBeAg, HBeAb, and HBcAb. The samples were further analyzed using the enzyme linked immunosorbent assay for HBsAg confirmation (Monolisa HBsAg ULTRA, Bio-Rad Ltd., Marnes-la-Coquette, France) in accordance with the manufacturer’s instruction. It is a one-step enzyme immunoassay technique of the sandwich type for the detection of the surface antigen of the hepatitis B virus (HBsAg) in human serum. All reagents were allowed to reach room temperature before running the assay.

#### 2.7.2. Biochemical Tests

Sera were subjected to liver function tests in order to determine the level of ALT, AST, ALP, albumin, and total and direct bilirubin. Kits used were that of Randox Laboratories Limited, (Crumlin, County Antrim, UK). Tests were carried out in accordance with the manufacturer’s instruction. Sera were allowed to reach room temperature before tests were run. The spectrometric method was used to determine the levels of ALT and AST as described by Reitman and Frankel [18]. The spectrometric method was also used to determine the level of ALP as described by Engelhardt [19], a bromocresol green method was used to determine the level of albumin as described by Grant et al. [20], and a colorimetric method was used for the determination of total and direct bilirubin as described by Jendrassik and Grof [21].

### 2.8. Data Analysis

Data obtained from test results were analyzed using IBM SPSS Version 20 statistical software (IBM Corp. Armonk, NY, USA). The relationship between HBV serologic markers and liver enzymes/compounds abnormalities were evaluated with binary logistic regression, where a *p*-value of less than 0.05 at 95% confidence interval (CI) was considered statistically significant.

## 3. Results

The overall prevalence of HBsAg in this study was 6.9% (Table 1). Out of the total sample screened, 149 (93.1%) were positive for at least one of HBV serological markers, while 6.9% were serologically negative for all HBV markers. None of the samples tested positive for HBeAg and each of HBsAb and HBeAb occurred only once (0.6%). As shown in Table 1, among the markers that occurred simultaneously, HBeAb and HBcAb occurred 71 times (44.4%), while HBsAb, HBeAb and HBcAb occurred only 10 times (6.3%). Table 1 also shows that, among those that tested positive for HBsAg only, 75%, 87.5%, 37.5%, and 37.5% had elevated levels of AST, ALP, and total and direct bilirubin, respectively. Out of the 50 patients that tested positive for HBcAb, 22%, 28%, and 4% had elevated levels of AST, ALP, and ALT, respectively. It was observed that most of the patients had normal levels of albumin. Among the patients that tested negative for all HBV markers, 63.6% had elevated levels of total and direct bilirubin, 27.3% had elevated levels of AST and ALP, while 18.2% had an albumin level below the reference limit.

As shown in Table 2 and Table 3, none of the participants consumes alcohol and only 2 out of the 160 participants had been vaccinated (Table 3).

**Figure 1:**
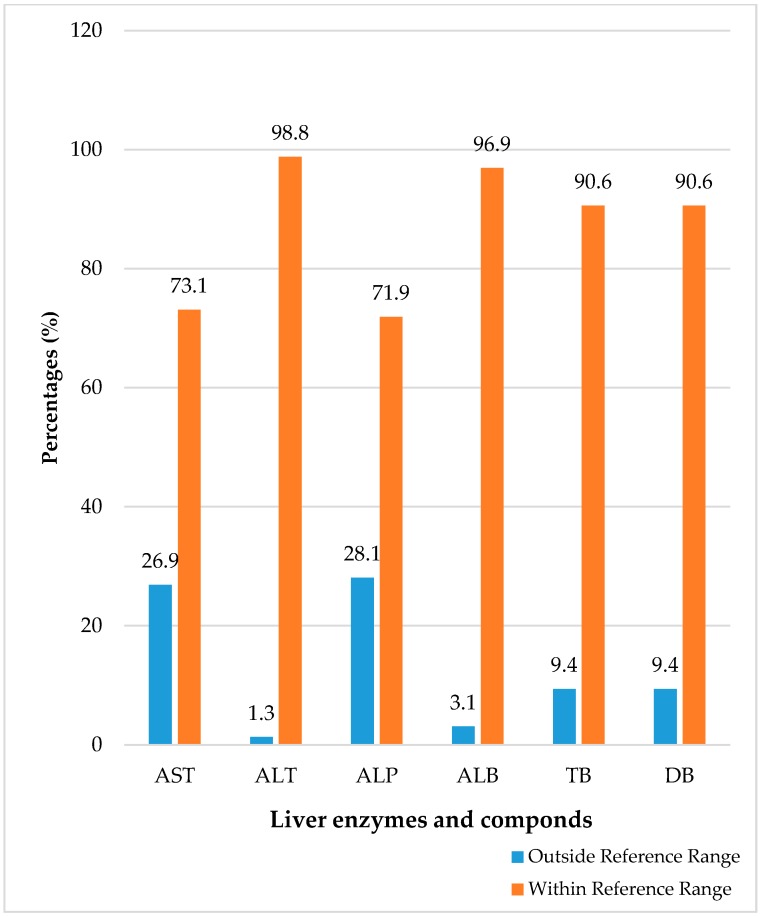
Distribution of liver enzymes and compounds among the pregnant women attending the antenatal clinics in the study area. AST: aspartate aminotransferase; ALT: alanine aminotransferase; ALP: alkaline phosphatase; ALB: albumin; TB: total bilirubin; DB: direct bilirubin.

As shown in Figure 1, most of the patients had their liver enzymes and compounds within the reference range. Out of the 160 serum samples subjected to liver function tests, 45 (28.1%) had elevated levels of ALP, 43 (26.9%) had elevated levels of AST, 15 (9.4%) had elevated levels of direct bilirubin, and only 2 (1.3%) had ALT levels above the reference limit.

As shown in Table 4, binary logistic regression was performed to ascertain if the abnormalities seen in the levels of liver enzymes and compounds was as a result of HBV infection. The predictors were significant at *p*-value (≤0.05). The model explained 43.7%, 4.8%, and 16.7% Nagelkerke *R*^2^ of HBsAg, HBeAb, and HBcAb, respectively. It also correctly classified 94.4% of the cases of HBsAg, 55.6% of the cases of HBeAb, and 82.5% of the cases of HBcAb. There was a statistically significant association between HBsAg seropositivity and an abnormal level of ALP (*p* = 0.02). The association was almost significant with AST and albumin abnormalities, as both *p*-values equal 0.06, but no significant association was observed between HBsAg seropositivity and ALT abnormality (*p* = 0.99). HBcAb seropositivity showed a statistically significant association only with abnormal albumin level (*p* = 0.04) but not with other liver enzymes and compounds. Additionally, HBeAb showed no significant association with AST (*p* = 0.84), ALT (*p* = 0.99), ALP (*p* = 0.38), albumin (*p* = 0.71), or total or direct bilirubin (*p* = 0.28) abnormalities.

## 4. Discussion

The HBsAg prevalence of 6.9% in this study was moderately high [22]. This is similar to the findings of the studies conducted in IdoEkiti (Ekiti State, Nigeria) [23], with a prevalence of 6.8%, and in Obudu (Cross River State, Nigeria) [24], with a prevalence of 6.6%. The presence of HBsAg in serum usually indicates recent HBV infection, although this marker can persist in chronic hepatitis [13]. Those with HBsAg are highly infectious. The absence of HBeAg in this study implies low levels of HBV viral replication and infectivity, while the presence of high prevalence of HBcAb shows that most of the participants have had hepatitis B infection in the past [10]. This study shows that none of the patients were alcohol consumers. This may be due to the fact that alcohol consumption is prohibited in Kano State, Nigeria, which is predominantly an Islamic state. Therefore, the alteration observed in the levels of these enzymes is not as a result of alcohol consumption, although an alcohol test was not carried out on the serum samples. The study also shows that only 1.3% of the participants had been vaccinated against HBV. This implies that vaccination coverage rate is low in the study area. This explains why the prevalence of past and present HBV infection is high among the study group.

The study also revealed that few of the participants (1.3%) with normal ALT level that tested positive for both HBsAg and HBeAb simultaneously, but negative for HBeAg were in their immune control phase (inactive carrier state) of chronic hepatitis. This indicates an early convalescence when HBeAg has declined below detectable levels. This phase is characterized by seroconversion from HBeAg to HBeAb positivity and normal ALT levels [25]. Elevation of liver enzymes was defined as a value above the upper reference limit of the reference laboratory where the tests were carried out. The same criterion was used for bilirubin. In albumin, value that falls below the normal reference range was termed abnormal. In viral hepatitis, the ratio of AST to ALT has more clinical utility than assessing individual elevated levels; the AST/ALT ratio (with elevated levels of both enzymes) is usually approximately 1:1. Besides, AST is less specific for liver disease. In many cases of liver inflammation, the ALT and AST levels are elevated together [26]. It seems that the lower percentage of abnormal ALT in this study may be due to the absence of HBeAg markers in all serum samples tested. Most previous studies with a high frequency of HBeAg seropositivity also have a high frequency of abnormal ALT. In a similar study conducted by Koki et al. [27] to show the activity of liver enzymes among HBV-positive patients, abnormal ALT and AST was observed among HBsAg and HBeAg-seropositive groups. However, abnormal ALP was not found to be significantly different between HBsAg and HBeAg seropositivity against HBsAg-positive and HBeAg-negative groups. They also found that the rate of abnormal ALT (71.0%) was higher than that of abnormal AST (58%) and abnormal ALP (32.30%) in HBsAg (+) subjects. In the work reported by Taura et al. [28], on the analysis of the liver enzyme activity in 200 HBsAg-seropositive patients, 17.0% were seropositive for HBeAg with elevated serum level of ALT and 44.8% of the HBeAg-infected subjects had ongoing liver damage, with 5% linked to HCC. 

This study shows that HBsAg seropositivity is significantly associated with an abnormal ALP level (*p* < 0.05), while HBcAb seropositivity showed a significant association with an abnormal albumin level (*p* < 0.05). This contrasts with the findings of Saleh [29] who found no significant differences in the levels of ALT, AST, and total and direct bilirubin between those with and those without HBsAg. However, the findings of Onwuliri et al. [30] show that there was a significant increase in the levels of AST and ALT between HBsAg-positive and -negative pregnant subjects. In a similar study conducted by Bayo et al. [31], there was no statistically significant difference between HBsAg-positive women and those with negative test results with respect to median values of liver enzymes.

It is very likely that some of the abnormalities in hepatic enzymes and compounds observed among these patients are due to HBV, since there are a large percentage of those that have been exposed to the virus in the past. In fact, few abnormal ALT levels (4%) were observed among those that tested positive for HBcAb only. This set of participants also had elevated levels of AST, ALP, and bilirubin. However, the pattern of distribution of these enzymes and compounds among most HBsAg-seropositive patients in this study suggests that these abnormalities may be a result of other conditions. This is because, among the 6.9% participants that were seronegative for all HBV markers, a large percentage also had abnormal levels of total and direct bilirubin, while few others had abnormal levels of AST and albumin. This clearly shows that the abnormal hepatic enzymes and compounds are not always caused by HBV infection. Furthermore, the abnormal levels of ALP and albumin observed in the study may be a result of pregnancy as well. Likewise, other conditions that may lead to hepatic enzymes elevation such as HCV cannot be ruled out. This study is not without limitations, as it did not consider trimester of pregnancy, medications used currently or previously, herbal or alternative remedies, or occupational exposure to toxins by subjects prior to this study, which may have effects on liver enzymes and compound level alteration. Additionally, we did not test for other viral hepatitis, serum alcohol, and HBV DNA serum level. However, every subject who tested positive for HBsAg was advised to visit a competent physician for consultation.

## 5. Conclusions

Alteration in hepatic enzymes and compounds may be a result of HBV infection, but most of the abnormalities of liver enzymes and compounds observed among the study group were caused by conditions other than HBV infection, such as pregnancy, because of the pattern of distribution. Therefore, HBV infection is not a good predictor of hepatic enzymes and compound level alterations during pregnancy. Few subjects were in an inactive carrier phase of chronic hepatitis and thus have the propensity to revert back to HBeAg, which may reactivate HBV replication and infectivity. It also seems that a marked elevation in ALT level depends on the presence of HBeAg in the serum. It is recommended that all cases of newly acquired HBV infection in pregnancy should be promptly managed by health care providers. In order to ascertain if there is damage to the liver due to HBV infection, liver function tests should be carried out on HBsAg-positive individuals. 

## Figures and Tables

**Table 1:** Distribution of different combinations of hepatitis B virus (HBV) markers in relation to liver enzymes and compounds alteration among the study group (*n* = 160).

Serological Markers	Frq (%)	URLAST (%)	URLALT (%)	URLALP (%)	URLTB (%)	URLDB (%)	LRLAlbumin (%)
HBsAg (+) only	8(5)	6 (75.0)	0 (0.0)	7 (87.5)	3 (37.5)	3 (37.5)	1 (12.5)
HBcAb (+) only	50(31.3)	11 (22.0)	2 (4.0)	14 (28.0)	3 (6.0)	3 (6.0)	0 (0.0)
HBsAg (+) and HBeAb (+)	2(1.3)	1 (50.0)	0 (0.0)	2 (100.0)	1(50.0)	1 (50.0)	1 (50.0)
HBeAb (+) and HBcAb (+)	71(44.4)	16 (22.5)	0 (0.0)	14 (19.7)	4 (5.6)	4 (5.6)	1 (1.4)
HBsAg (+), HBeAb (+) and HBcAb (+)	1(0.6)	1 (100.0)	0 (0.0)	1 (100.0)	1 (100.0)	1 (100.0)	0 (0.0)
HBsAb (+), HBeAb (+) andHBcAb (+)	10(6.3)	2 (20.0)	0 (0.0)	2 (20.0)	1 (10.0)	1 (10.0)	1 (10.0)
Seronegative	11(6.9)	3 (27.3)	0 (0.0)	3 (27.3)	7 (63.6)	7 (63.6)	2 (18.2)
SamplesHBsAg (+) Overall	11(6.9)	8 (72.7)	0 (0.0)	8 (72.7)	4 (36.7)	4 (36.7)	1(9.1)

(+): seropositivity; URL: upper reference limit; LRL: lower reference limit; Frq: frequency.

**Table 2:** Alcohol consumption among the study group (*n* = 160).

Alcohol Consumption	Count	Percentage
+	0	0.0
-	160	100.0

(+): those that consume alcohol.; (-): those that do not consume alcohol.

**Table 3:** HBV vaccination status of the study group (*n* = 160).

Vaccination Status	Count	Percentage
+	2	1.3
-	158	98.8

(+): those that have been vaccinated with HBV vaccine; (-): those that have not been vaccinated with HBV vaccine.

**Table 4:** Predictors of liver enzymes and compounds abnormalities in relation to HBV infection among the pregnant women attending clinics in the study area.

Marker	B	SE	Wald	df	*p*-Value
**AST**					
HbsAg	1.62	0.87	3.43	1	0.06
HbeAb	0.08	0.40	0.03	1	0.84
HbcAb	−0.86	0.49	3.02	1	0.82
**ALT**					
HbsAg	−20.09	28,420.67	0.00	1	0.99
HbeAb	−21.31	28,419.89	0.00	1	0.99
HbcAb	20.49	28,420.53	0.00	1	0.99
**ALP**					
HbsAg	2.69	1.12	5.75	1	0.02 *
HbeAb	−0.35	0.41	0.74	1	0.38
HbcAb	−0.69	0.50	1.88	1	0.17
**TB/DB**					
HbsAg	0.94	0.81	1.34	1	0.24
HbeAb	−0.62	0.58	1.13	1	0.28
HbcAb	−0.76	0.63	1.43	1	0.23
**Albumin**					
HbsAg	2.47	1.31	3.51	1	0.06
HbeAb	0.35	0.96	0.13	1	0.71
HbcAb	−1.97	1.00	3.88	1	0.04 *

*p*-Value significant at ≤0.05 and represented with *; *p*-value insignificant at ≥0.05; confidence interval (CI) at 95%. Negelkerke *R*^2^ for HBsAg, HBeAb, and HBcAb are 43.7%, 4.8%, and 16.7%, respectively. Classification cases for HBsAg, HBeAb, and HBcAb are 94.4%, 55.6%, and 82.5%, respectively. B: regression co-efficient; df: degree of freedom; SE: standard error; wald: ratio of the square of the B to SE.
